# Uterine Involution and Reproductive Performance in Dairy Cows with Metabolic Diseases

**DOI:** 10.3390/ani9030093

**Published:** 2019-03-18

**Authors:** Renan Braga Paiano, Daniela Becker Birgel, Eduardo Harry Birgel Junior

**Affiliations:** 1 Department of Anatomy of Domestic and Wild Animals, School of Veterinary Medicine and Animal Sciences, University of São Paulo, São Paulo 05508270, Brazil; ehbirgel@usp.br; 2 Department of Reproduction Animal, School of Veterinary Medicine and Animal Sciences, University of São Paulo, São Paulo 05508270, Brazil; 3 Department of Veterinary Medicine, College of Animal Science and Food Engineering, University of São Paulo, Pirassununga 13635900, Brazil; dabirgel@usp.br

**Keywords:** lactating cows, hypocalcemia, lipomobilization, hyperketonemia, uterus

## Abstract

**Simple Summary:**

Metabolic diseases, such as hypocalcemia, ketosis and lipomobilization, cause financial losses in dairy farms, mainly due to the costs of treatment, milk discharges and decreased milk production of diseased animals. Four groups of dairy cows were selected. The uterine involution of the animals was evaluated by palpation and transrectal ultrasonography during the postpartum period. In conclusion, cows with metabolic diseases had a delayed uterine involution when compared to animals without metabolic diseases.

**Abstract:**

The aim of this study was to investigate the effects of metabolic diseases on uterine involution and reproductive performance during the postpartum period. Multiparous Holstein dairy cows (*n* = 50) were divided into four groups based on whether they were healthy (*n* = 14), or had lipomobilization (*n* = 14), hypocalcemia (*n* = 11), and hyperketonemia (*n* = 11). Transrectal palpation and transrectal B-Mode sonography were carried out on days 7, 14, 21, 30, 45 and 60 after parturition. Cows with metabolic disease had a greater (*p* < 0.05) uterine size as assessed transrectally compared with cows without metabolic disease. Sonographic measurements revealed a greater (*p* < 0.05) horn diameter and endometrial thickness in cows of the metabolic disease groups than in the healthy cows. Metabolic disease affected (*p* < 0.05) the milk yield, percentage of service per pregnancy, days to first ovulation and days open. In conclusion, metabolic disease affected the uterine involution and fertility during the postpartum period.

## 1. Introduction

During the transition period, dairy cows undergo a period of physiological adaptations marked by the increase in energy demand to meet milk production demands. This is associated with reduced food intake, and contributes to the beginning of a negative energy balance (NEB) [1,2,3,4]. An intense NEB can cause a depression of the immune system, due to the impairment of leukocyte function, favoring changes in the metabolic profile, which can impair the health of the animals and increase the risk of developing metabolic diseases such as lipomobilization, ketosis and hypocalcemia [5,6,7,8,9,10]. Metabolic diseases can cause negative effects on the reproductive performance of dairy cows, which can increase the period of calving up to the first insemination, open days, the number of services per conception, reduce conception rate and cause a higher prevalence of puerperal disorders such as metritis and endometritis [11,12,13].

During this period, which occurs after calving, dairy cows undergo profound physiological changes involving the regeneration of the uterine tract, including endometrial regeneration, necrosis, and relaxation of the vulva, vagina and cervix, which can facilitate the entry of bacteria that can contaminate the uterine lumen [14,15]. During this period, the uterus of the cattle is contaminated by environmental microorganisms, with about 90% of cows presenting contamination during the first two weeks after parturition. However, the longevity of these bacteria in the uterine environment can cause uterine diseases [14,16,17]. It has been reported that uterine involution is delayed in dairy cows with puerperal diseases [18,19]. Several studies have shown that dairy cows that develop metabolic disorders have an increased incidence of diseases during the puerperium period [20,21,22,23]. However, to our knowledge, no papers have demonstrated that uterine involution is delayed in dairy cows with metabolic disorders. Thus, the main objective of the present study was to investigate the effects of metabolic diseases, including hyperketonemia, lipomobilization and hypocalcemia, on uterine involution, reproductive performance and the prevalence of metritis and endometritis during the postpartum period.

## 2. Materials and Methods

### 2.1. Ethical Statement

All animal procedures were approved by the Bioethics Committee of the School of Veterinary Medicine and Animal Sciences, University of São Paulo, São Paulo, Brazil (8022150216/2018).

### 2.2. Animals

The study was conducted at the Dairy Farm of the University of São Paulo, Fernando Costa Campus, Pirassununga, Brazil, between February 2017 and October 2017. In total, 50 dairy cows, with the same number of lactations, age and body condition score (BCS), and with an average milk yield of 9800 kg in the previous lactation were used in this study. The cows were housed in a freestall barn. Cows had free access to a total mixed ratio (TMR) provided for ad libitum twice daily at 0800 and 1600 h. The close-up diet consisted of 69.48% of corn silage, 16.63% of ground corn, 8.94% of soybean meal, 0.94% of urea and 4.01% of anionic salt (Minerthal nucleus milk prepartum^®^, Minerthal, Brazil), and designed to contain 13.13% of crude protein, 4.09% of ether extract, and 37.59% of neutral detergent fiber on dry matter (DM) basis. Dry cows were moved from a far-off to a close-up pen with between 21–28 days before expected calving. The postpartum diet was formulated to contain 49.98% of corn silage, 26.77% of ground corn, 20.86% of soybean meal, 0.22% of urea, 0.29% of limestone, 0.22% of dicalcium phosphate, 0.36% of salt and 1.30% of mineral and vitamin salt (Minerthal milk MD^®^, Minerthal, Brazil), and designed to contain 17.07% of crude protein, 3.05% of ether extract, and 31.68% of neutral detergent fiber on dry matter (DM) basis. All calving cows were milked twice daily.

### 2.3. Study Design

The cows were divided into a group of healthy cows free of any metabolic disease (*n* = 14, mean lactation: 2.29 ± 0.18 and the BCS at day 21 before the parturition was 3.38 ± 0.20), a group of cows with lipomobilization (*n* = 14, mean lactation: 2.29 ± 0.20 and the BCS at day 21 before the parturition was 3.42 ± 0.19), a group of cows with hypocalcemia (*n* = 11, mean lactation: 2.34 ± 0.22 and the BCS at day 21 before the parturition was 3.42 ± 0.19), and a group of cows with hyperketonemia (*n* = 11, mean lactation: 2.33 ± 0.24 and the BCS at day 21 before the parturition was 3.43 ± 0.20). Lipomobilization was defined by a serum of non-esterified fatty acids (NEFA) concentration higher than 0.4 mmol/L in at least one of three samples at the points: −7, −4 and −2 two days before calving [24]. Hypocalcemia was characterized by <8.0 mg/dL calcium from 3 to 8 h after delivery [25]. Hyperketonemia was established by a β-hydroxybutyric acid (BHBA) serum concentration >1200 µmol/L in at least one of three samples at the points: Parturition, +1 and +7 days postpartum [26]. Cows diagnosed with lipomobilization, hypocalcemia and hyperketonemia had spontaneous delivery without obstetric assistance and no other concurrent metabolic diseases.

### 2.4. Blood Sampling and Analysis

To evaluate the metabolic alterations, blood samples were collected from all cows from coccygeal vein at 07:00 before feeding on −7, −4, and −2 days before parturition, at parturition (3–8 h after parturition), and +1, +7, +14, +21, +30, +45 and +60 days after calving. All blood samples were collected into evacuated tubes (Becton Dickinson Vacutainer Systems, Franklin Lakes, NJ, USA). Tubes were placed in ice until centrifugation and within 2 h of collection, blood samples were centrifuged at 2500× *g* for 20 min. Serum samples were stored at −20 °C until analysis measurements. The concentration of total BHBA, NEFA and calcium were determined using commercial kits from Randox in an automatic biochemistry system (RX Daytona—Randox Laboratories, Crumlin, UK).

### 2.5. Body Condition Score and Milk Yield

On days −7, −4, and −2 before calving, at parturition, and on days +1, +7, +14, +21, +30, +45 and +60 after parturition the body condition score was measured using the 5-point scale [27]. Milk yield was recorded at each milking and stored by computer software program (Alpro, DeLaval, Tumba, Sweden). Daily milk weights were extracted and used to calculate the average from 7 to 60 days postpartum.

### 2.6. Reproductive Evaluation

Monitoring of uterine involution was performed by transrectal palpation and by transrectal ultrasonography at the following times: 7, 14, 21, 30, 45 and 60 days after parturition. The size of the uterus was measured according to Grunert [28]: The uterus could be retracted and the uterine horn ≤ 2 cm (score 1), the uterus could be retracted and the uterine horn was 3–5 cm (score 2), the uterus could be retracted and the uterine horn was 6–8 cm (score 3), margins of the uterus can be delimited by the hand and the uterine horn was 9–20 cm (score 4), part of the uterus was not palpable or incompletely palpable (score 5), or margins of the uterus cannot be delineated by hand (score 6). All sonographic investigations were conducted using an M-Turbo ultrasonic machine (Sonosite Co., Bothel, NY, USA), equipped with a 7.5 MHz linear transducer. The diameters of pregnant and non-pregnant horns were evaluated as described by Heppelmann et al. [19]. The previously pregnant uterine horn was due its larger size relative to the non-pregnant horn. The position of the transducer for the examination was approximately 2 cm cranial to the bifurcation. Cross-sectional images were obtained by placing the probe in a transverse direction. The image was frozen and the mean of two measurements (transverse and longitudinal axis) was calculated. Endometrial thicknesses of pregnant and non-pregnant horns were measured using the internal calipers of the ultrasonography machine according to López-Helguera et al. [29]. Services per pregnancy was defined as the total number of inseminations divided by the number of pregnant cows. Days open were characterized by the days from calving to pregnancy.

Cows were evaluated for diagnoses of puerperal disease by transrectal palpation, including metritis, on days 7 and 14 postpartum. Metritis was characterized by an enlarged uterus with red-brown watery vaginal discharge, and a rectal temperature of ≥39.5 °C [30]. Endometritis was evaluated by vaginal palpation 28 days postpartum; the vulvar region was cleaned and then a gloved hand was introduced to collect the vaginal secretion. Subsequently, a sample of vaginal discharge was observed by direct inspection and classified based on a 3-pint scale according to Williams et al. [31]: 0 = Clear mucus, 1 = Clear discharge with pus flecks, 2 = Mucopurulent with 50% mucus and 50% pus, and 3 = More than 50% of pus. Any cow with a vaginal discharge score ≥2 was considered to have endometritis.

### 2.7. Statistical Analysis

The metabolic profile, BCS, milk yield and the size and diameter of the uterus and endometrial thickness were analyzed by the GLIMMIX procedure of SAS (SAS Institute Inc., Cary, NC, USA, Version 9.3). PROC GLIMMIX was used to analyze the number of inseminations per pregnancy, days to first ovulation and days open. The mean parameters of the different groups were compared using a one-way analysis of variance. Tukey’s test was used to detect differences among means. The effects of metabolic diseases on the incidence of metritis and endometritis, pregnancy and pregnancy loss rates were analyzed by logistic regression using PROC GLIMMIX, fitting a binary distribution. *p* < 0.05 was considered to be statistically significant.

## 3. Results

**Figure 1:**
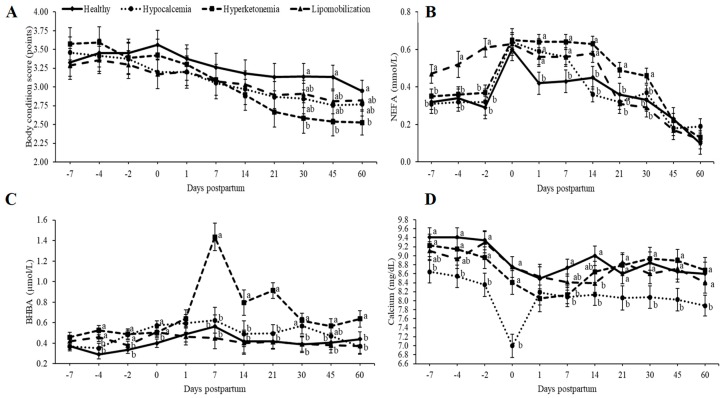
(**a**) Body condition score; (**b**) NEFA (non-esterified fatty acids); (**c**) BHBA (β-hydroxybutyric acid) and (**d**) calcium (least squares means ± SEM) in relation to days postpartum. ^a,b,c^ Different superscript letters differ at *p* < 0.05.

The data presented in Figure 1 allow for the characterization of the metabolic disturbances of the animals classified with lipomobilization, hyperketonemia and hypocalcemia. Cows with hyperketonemia showed lower (*p* < 0.05) BCS on days 30, 45 and 60 compared to healthy cows (Figure 1). Based on metabolic profile, the cows in the hyperketonemia group (on days 1, 7, 14, 21, and 30 after parturition), lipomobilization group (on days 7, 4 and 2 before calving and days 1, 7 and 14 after parturition) and hypocalcemic group (on days 1 and 7 after calving) had higher (*p* < 0.05) NEFA than cows without metabolic disease (Figure 1). The BHBA concentration was higher (*p* < 0.05) for cows with hyperketonemia (on days 4 and 2 before calving, at parturition, and on days 7, 14, 21, 30, 45 and 60), lipomobilization group (on day 4 before calving and at parturition), and hypocalcemic group (on day 2 before parturition, at parturition, and on day 30 after calving) had lower (*p* < 0.05) NEFA than cows without metabolic disease (Figure 1). The calcium concentration was lower (*p* < 0.05) for the hyperketonemic group (on day 7), lipomobilization group (on day 14) and hypocalcemic group (on days 7, 4, and 2 before calving, at parturition and on days 7, 14, 21, 30, 45 and 60) than healthy cows (Figure 1).

**Figure 2:**
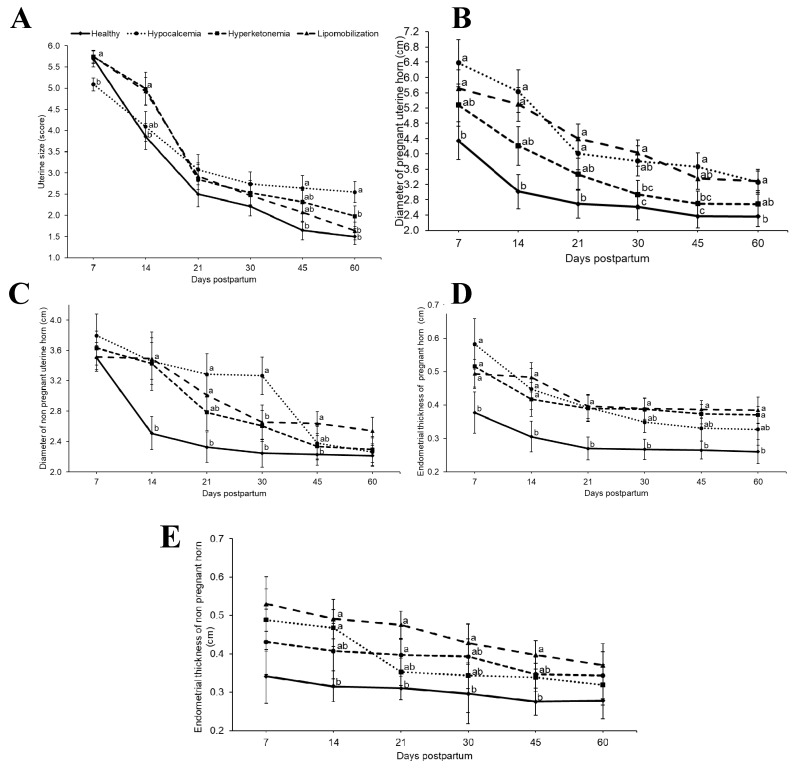
(**a**) Uterine size; (**b**) diameter of pregnant uterine horn; (**c**) diameter of non-pregnant horn; (**d**) endometrial thickness of pregnant horn; (**e**) endometrial thickness of non-pregnant horn (least squares means ± SEM) in relation to days postpartum. ^a,b,c^ Different superscript letters differ at *p* < 0.05.

In terms of uterine involution, the size and diameter of uterine horns and endometrial thickness differed between groups (*p* < 0.05, Figure 2). For uterus size, evaluated by transrectal palpation, cows with hyperketonemia and lipomobilization had greater values at day 14 postpartum, whereas hypocalcemia group showed greater values than healthy cows on days 45 and 60 (*p* < 0.05). Based on the ultrasound evaluation, the diameter of previously pregnant uterine horn was greater in cows with hypocalcemia (on days 7, 14, 21, 30, 45 and 60) and lipomobilization groups (on days 7, 14, 21, 30, 45 and 60), compared to the diameters of healthy cows (*p* < 0.05). As for the diameter of the non-pregnant uterine horns, hypocalcemia (on days 14, 21 and 30), hyperketonemia (on day 14) and lipomobilization group cows (on days 14, 21, and 45) had larger values than healthy cows (*p* < 0.05). For endometrial thickness of previously pregnant uterine horn, the lipomobilization, hyperketonemia (both on days 7, 14, 21, 30, 45 and 60) and hypocalcemia groups (on days 7, 14, 21 and 30) showed higher values than cows without metabolic disease (*p* < 0.05). For the endometrial thickness of the non-pregnant uterine horn, the lipomobilization (on day 14), hyperketonemia (on days 14, 21, 30 and 45) and hypocalcemia groups (on days 7, 14, 21 and 30) had higher values than cows without metabolic disease (*p* < 0.05).

Milk yield differed among experimental groups (*p* < 0.05). Cows with hypocalcemia (23.17 kg), hyperketonemia (20.16 kg), and lipomobilization (20.18) produced less milk than healthy cows (29.41) (Table 1). There was no difference among groups in terms of the incidence of metritis and endometritis (Table 1). The percentage of services per pregnancy was lower (*p* < 0.05) for healthy cows (2.14) than for cows with hypocalcemia (3.18) (Table 1). Days to first ovulation and days open (Table 1) differed between the groups (*p* < 0.05), with healthy cows having fewer (*p* < 0.05) days open (124.14) than cows with hypocalcemia (164.73), hyperketonemia (164.82) and lipomobilization (160.50). The number of the days to the first ovulation was lower (*p* < 0.05) for healthy cows (25.57) than for cows with hyperketonemia (45.45) and lipomobilization (42).

## 4. Discussion

Cows with metabolic disease may have reduced uterine contractility, impairing the elimination of the lochia, affecting the function of neutrophils, and causing losses in fertility during the postpartum period [25,32,33,34]. Our data indicate that the metabolic disease delayed the process of uterine involution.

According to the diameter of the uterine horn, obtained by ultrasound examination, the observed values for the previously pregnant uterine horn of animals with hypocalcemia and lipomobilization were higher than those described by Mateus et al. [18] for cows with endometritis. Heppelmann et al. [19] noted a larger diameter of the formerly pregnant uterine horn in cows with uterine disease 11 days postpartum than that of cows without uterine disease; their values were higher than those of our cows with hypocalcemia, hyperketonemia and lipomobilization during the first two weeks after parturition. The diameters of the pregnant and non-pregnant uterine horns for cows with hypocalcemia, hyperketonemia and lipomobilization were higher than those found by Saut et al. [35] who evaluated the uterine involution of dairy cows during the puerperium period. In this study, endometrial thickness of the previously pregnant and non-pregnant uterine horns was lower than that described by López-Helguera et al. [29] who assessed the influence of the genital tract status on the fertility of high-yield dairy cows.

To date, there have been no studies describing the influence of metabolic diseases on involution using ultrasonography. These results indicate that cows that had metabolic diseases had a greater uterine horn diameter than those described for animals with puerperal diseases, demonstrating that metabolic disorders can cause a greater delay in uterine involution than uterine diseases. 

Cows with metabolic diseases (e.g., ketosis, milk fever, and fatty liver) are more susceptible to develop puerperal diseases. A connection between metabolic diseases, uterine involution and reproductive performance might be an innate immune response or acute phase response. Dairy cows affected by metabolic diseases show alterations in innate immunity during the dry off period and the postpartum period [9,36,37,38]. Serum interleukin-1, interleukin-6, tumor necrosis factor, haptoglobin, serum amyloid A and lactate were increased in cows with metabolic diseases from −8 and −4 weeks before calving, as well as during and after diagnosis of the disease [9,36,37,38]. 

As lipomobilization, ketosis, hypocalcemia and innate immune response are associated; this may interfere in the evolution of the puerperium, predisposing to the onset of diseases such as metritis, endometritis. Chapinal et al. [21], who studied the association between serum NEFA and disease during the transitional period, found that prepartum cows with NEFA values >0.3 mmol/L were 1.8 times more likely to develop metritis. Ospina et al. [24] related that the NEFA values >0.3 mmol/L from 1 to 2 weeks before the parturition is associated with an increased risk of retained placenta, abomasal displacement, and metritis during the puerperium. Duffield et al. [39] found that cows with BHBA values >1.20 mmol/L in the first week postpartum were 3 times more likely to develop metritis. Shin et al. [40] showed that animals with ketosis had a higher prevalence of endometritis (44.1%) when compared to non-ketotic animals (26.7%). Reduction in the calcium concentration contributes to an increased incidence of dystocia, retained placenta, metritis, endometritis, mastitis and displacement of abomasum [22,32,34,41,42].

In terms of the prevalence of uterine diseases, no difference was noted in our data between animals with and without metabolic diseases, but our data indicates that a delay in uterine involution was associated with a delay in the return to cyclicity for cows with hyperketonemia and lipomobilization and an increase in the number of services per pregnancy for cows with hypocalcemia. Cows that had metabolic diseases (hypocalcemia, hyperketonemia and lipomobilization) had a greater number of open days to become pregnant. 

The values described by Barletta et al. [43] for the number of days until first ovulation in animals that lost body condition score during the transition period (47.1 d) was higher than those observed in this study for animals with hypocalcemia (35.18 d), hyperketonemia (45.45 d) and lipomobilization (42 d). Shin et al. [40] reported a conception pregnancy rate after the first artificial insemination of 28.1% for animals with ketosis. This value was lower than that observed for the animals in this study with hypocalcemia (36.36%), but similar to that observed for the group of cows with hyperketonemia (27.27%) and lipomobilization (28.57%). Plöntzke et al. [44] and Barrio et al. [45] noted that animals with endometritis were open for 160 and 154 days, respectively; these values were similar to those observed in this study for animals with hypocalcemia (164.73 d), hyperketonemia (164.82 d) and lipomobilization (160.5 d). Plöntzke et al. [44] described that cows with endometritis required 3.7 services per pregnancy, which is higher than in this study for cows with hypocalcemia (3.18), hyperketonemia (2.63) and lipomobilization (2.71). Chapinal et al. [21] observed a negative effect of NEFA on pregnancy at the first artificial insemination, and a positive correlation between hyperketonemia and an increased interval between calving to conception [46]. A high concentration of BHBA during the postpartum period has been associated with reduced pregnancy after the first insemination [47]. Cows with a NEFA serum concentration >0.3 mmol/L during prepartum had a lower pregnancy rate at first insemination [48]. Cows with serum BHBA values >1.0 mmol/L during the first seven days after delivery have a 1.5-fold higher chance of being anovular at week 9 postpartum [49], and have a lower pregnancy rate at 140 days in milk [47]. 

The low reproductive outcomes in animals with metabolic diseases can be justified as a consequence of decreased contractility of the uterus in the early puerperium. Cows with hypocalcemia may exhibit reduced muscle contraction, which results in reduced motility of the digestive organs including the abomasum and rumen, in addition to reproductive organs such as the uterus [25]. The low reproductive performance may be associated with the fact that animals with blood metabolite disturbances may have alterations in the composition of follicular fluid, which may impair follicular steroidogenesis and oocyte development, compromising cyclic resumption and fertility [22,50]. Another possibility may be related to failures in the innate immune response associated with the existence of metabolic diseases [36,37], making the uterine clearance process slower, determining the increase in endometrial thickness and uterine horn size.

As was expected according to these data, it was shown that cows with metabolic diseases produced less milk than healthy cows, showing that besides impairing the fertility of animals, metabolic diseases can also affect the productivity of the herd. Lower milk yield was also observed by Zhang et al. [38] in cows with hypocalcemia and Zhang et al. [9] in cows with hyperketonemia when compared to the cows in the control group.

## 5. Conclusions

Metabolic diseases have negative effects on uterine involution. Furthermore, according to our data, cows with metabolic diseases show impaired fertility and productivity during the postpartum period. 

## Figures and Tables

**Table 1:** Effect of metabolic disease during peripartum on milk yield, prevalence of metritis and endometritis, and service per pregnancy, day of first ovulation and days open.

Item	Cows without Metabolic Diseases	Hypocalcemia	Hyperketonemia	Lipomobilization
Milk yield, kg/d ± SD	29.41 ± 2.10 ^a^	23.17 ± 3.14 ^b^	20.16 ± 3.24 ^b^	20.18 ± 3.63 ^b^
Metritis, % (n)	21.43 (3/14)	36.36 (4/11)	27.27 (3/11)	42.86 (6/14)
Endometritis, % (n)	14.29 (2/14)	36.36 (4/11)	27.27 (3/11)	35.71 (5/14)
Service per pregnancy, mean ± SD	2.14 ± 0.19 ^b^	3.18 ± 0.22 ^a^	2.63 ± 0.22 ^ab^	2.71 ± 0.19 ^b^
First ovulation day, mean ± SD	27.57 ± 3.53 ^b^	35.18 ± 3.99 ^ab^	45.45 ± 3.99 ^a^	42.00 ± 3.53 ^a^
Days open, mean ± SD	124.14 ± 7.67 ^b^	164.73 ± 8.66 ^a^	164.82 ± 8.66 ^a^	160.50 ± 7.67 ^a^

^a,b^ Values within a row with different superscript letters differ at *p* < 0.05.
